# Energy Alignment of Quantum-Confined ZnO Particles with Copper Oxides for Heterojunctions with Improved Photocatalytic Performance

**DOI:** 10.1021/acsnanoscienceau.1c00040

**Published:** 2021-12-21

**Authors:** Jakob Thyr, José Montero, Lars Österlund, Tomas Edvinsson

**Affiliations:** Department of Materials Science and Engineering, Division of Solid State Physics, Uppsala University, P.O. box 35, SE 75103 Uppsala, Sweden

**Keywords:** quantum confinement, ZnO quantum dots, copper oxide, energy alignment, heterojunctions, photocatalysis

## Abstract

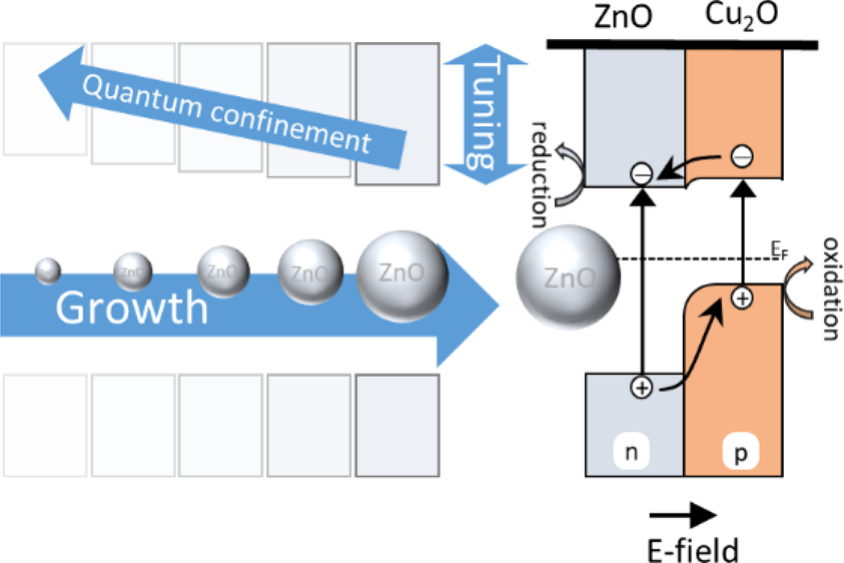

The ability to control electronic states by utilizing quantum confinement of one of the material components in heterojunctions is a promising approach to perform energy-level matching. In this work, we report the possibility to achieve optimum energy alignment in heterojunctions made from size-controlled quantum dots (Q-dots) of ZnO in combination with three copper oxides: Cu_2_O, Cu_4_O_3_, and CuO. Quantum confinement effects on the ZnO nanoparticles in the diameter range 2.6–7.4 nm showed that the direct optical band gap decreased from 3.99 to 3.41 eV, with a dominating shift occurring in the conduction band (CB) edge, and thus the possibility to obtain close to 0.6 eV CB edge shift by controlling the size of ZnO. The effect was utilized to align the electronic bands in the ZnO Q-dot/copper oxide heterojunctions to allow for charge transfer between the materials and to test the ability to improve the photocatalytic performance for the system, evaluated by the transformation of a dye molecule in water. The catalyst materials were investigated by X-ray diffraction, scanning electron microscopy, ultraviolet–visible (UV–vis), photoluminescence, and Raman spectroscopy. The most promising material combination was found to be the Cu_2_O copper oxide in combination with an energy aligned ZnO Q-dot system with approximately 7 nm diameter, showing strong synergy effects in good agreement with the energy-level analysis, outperforming the added effect of its individual components, ZnO-Q-dots and Cu_2_O, by about 140%. The results show that utilization of a heterojunction with controllable energy alignment can provide a drastically improved photocatalytic performance. Apart from increased photocatalytic activity, specific surface states of ZnO are quenched when the heterojunction is created. It is anticipated that the same approach can be utilized in several material combinations with the added benefit of a system with controllable overpotential and thus added specificity for the targeted reduction reaction.

## Introduction

**Figure 1:**
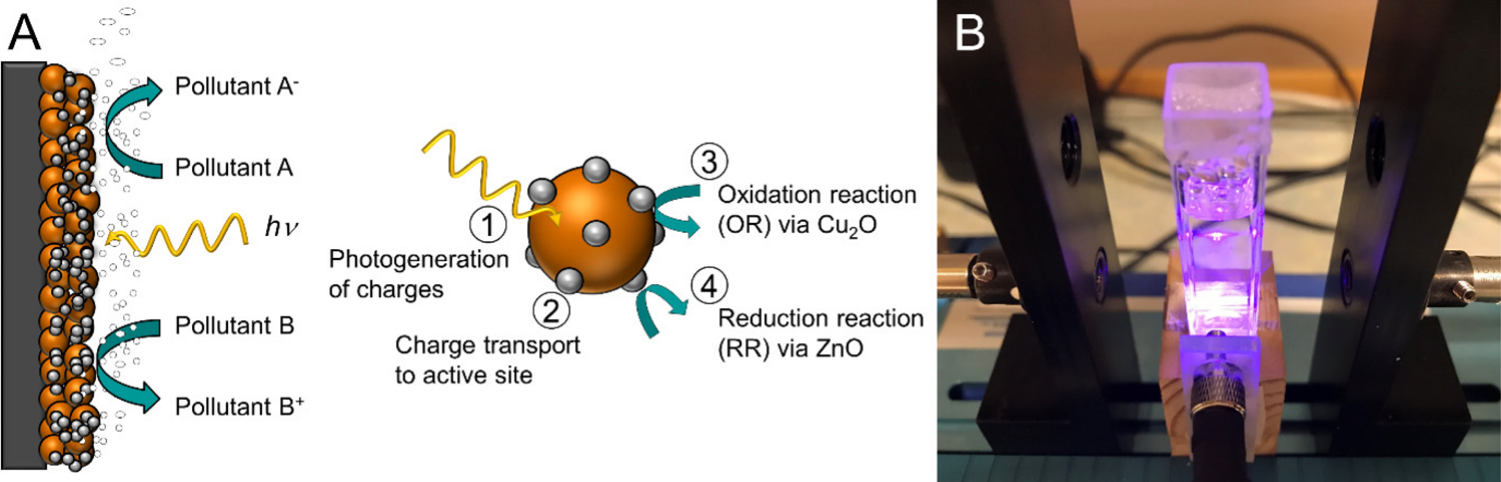
(A) Schematic illustration of key processes in photocatalytic reactions using the ZnO Q-dot/Cu_2_O system, one of the ZnO–copper oxide systems investigated in this study, and (B) a photograph of the experimental setup of an illuminated sample in the photocatalytic dye degradation experiment.

The importance of water can hardly be exaggerated. It is a prerequisite of life on earth and a resource that is needed in vast amounts for agriculture, industry, and domestic use.^1^ Water availability, however, is unevenly distributed across the world, and several densely populated regions are now subject to water scarcity.^1,2^ Water can also be used for energy generation and irrigation, and water, energy, and food production are all interdependent. This complex intertwined relation is called the water-energy-food nexus^3^ and leads to a situation where the demand for water, energy, and food competes for the available water resources. Water pollution from cities or factories together with an increased demand for freshwater due to the growing world population has strongly increased the pressure on available resources of clean water, evidenced in geopolitical competition of water resources.^4,5^ According to the World Health Organization (WHO) and UN-water, there are currently over 2.2 billion people lacking access to safely managed water.^6^ In many areas, arid climate is combined with economic water scarcity and lack of infrastructure to extract, manage, and distribute water. In such locations, demands on technical water purification methods are high. They need to be simple, freestanding, and inexpensive. Here, photocatalytic water purification using metal oxides match these demands as a final disinfection step, but has so far been limited by low efficiency. We, here, devise a photocatalytic system based on zinc oxide Q-dots and copper oxides, inexpensive raw materials. The energy needed for the purification process in photocatalytic water-cleaning technologies can be provided by sunlight, enabling off-grid solutions with no need for chemical disinfectant supply or maintenance. Photocatalytic degradation can be rationalized into a number of subsequent steps: photogeneration of charges, charge transport, and the reduction and oxidation reaction at the catalyst surface (Figure 1). The degradation of pollutants occurs by redox reactions at or near the surface, initiated by reactive oxygen species (ROS) created at the surface in aerated water.

The efficiency of photocatalysis is an issue that has attracted much attention. Several materials with good catalytic properties, like TiO_2_, ZnO, and SnO_2_ are limited by their high band gap and absorption only in the ultraviolet (UV) range,^7−10^ whereas others, like Cu_2_O, have a high charge recombination, which results in a short diffusion length, preventing the charges from reaching the material surface.^11^ In this study, we show that by combining two different photocatalysts in a heterojunction, we can improve the catalyst properties in both these aspects as well as add the possibility to control the energy alignment using quantum confinement effects. Depositing an UV-absorbing ZnO Q-dot layer on the top of a vis-absorbing Cu_*x*_O_*y*_ surface creates a Z-scheme-like structure that absorbs a large part of the solar light. Using one n-type material and one of the p-type materials, as in the case of ZnO and copper oxide, one creates a pn-heterojunction that induces a local electric field, facilitating electron-hole pair separation and increase in the efficiency of the photocatalytic process compared to the standalone materials. To form an efficient pn-heterojunction, the electronic levels of the materials should be appropriately positioned relative to each other, where energy alignment remains a challenge in nanomaterial science. One option is to change the material class, another is to choose different crystalline phases of the same material class, as demonstrated here with different copper oxides (Cu_2_O, Cu_4_O_3_, and CuO). A less-explored option is to utilize quantum confinement, where the change in the optical band gap implicitly means shift of the conduction band (CB) edge, the valence band (VB) edge, or both. This is utilized here for size-controlled ZnO, where the major shift occurs at the CB edge and utilized to energy-align the ZnO Q-dot system with the most suitable copper oxide to form an np-heterojunction with efficient charge separation and transfer of the photogenerated charge carriers. The ZnO–copper oxide material system possesses unique possibilities for controlling the electronic levels: For copper oxide, the different oxidized phases can be utilized, and for ZnO, one can use the quantum confinement effects in quantum crystals. The quantum-confined crystals can be viewed as an intermediate between a molecular state and a bulk material and by controlling the size of the crystals, which gives the degree of quantum confinement, the electronic states in the material can be tuned.

## Results and Discussion

**Figure 2:**
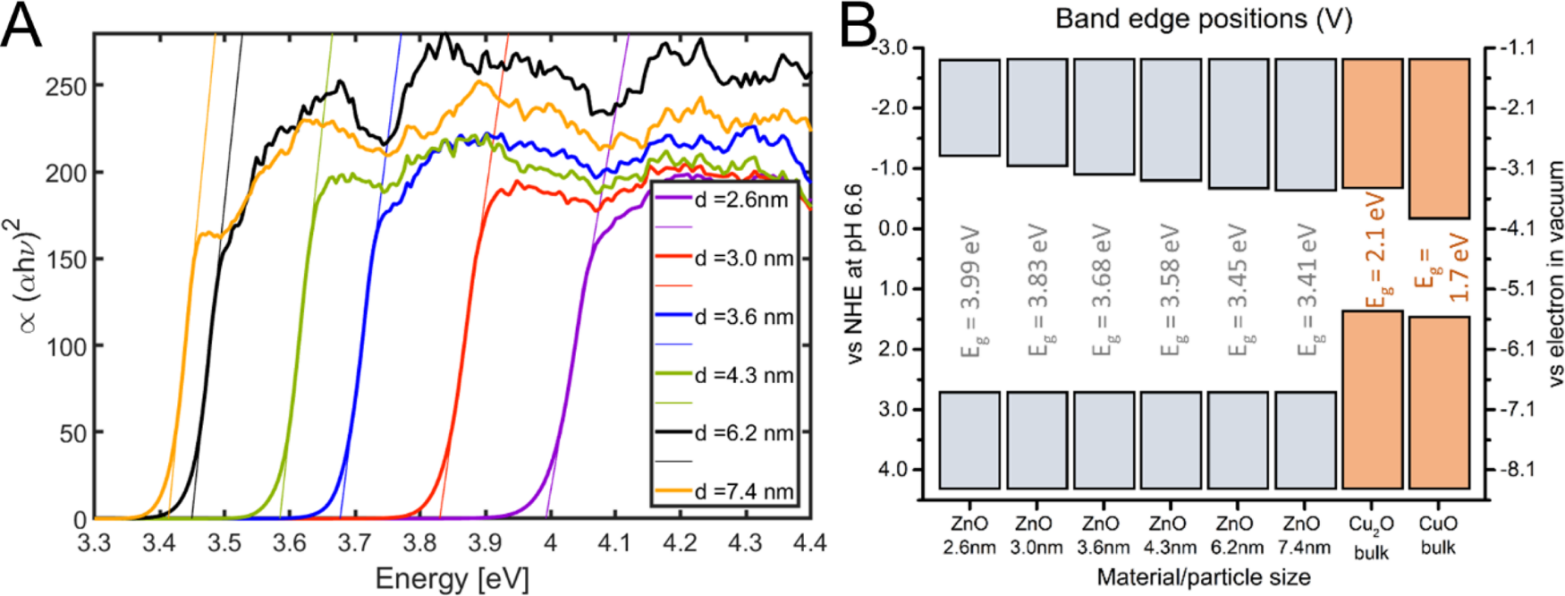
(A) Tauc plot showing how the band gap diminishes as the ZnO Q-dots grow in the solution during the synthesis and (B) CB and VB positions at pH 6.6 for six different sizes of ZnO Q-dots and bulk cuprous oxide and cupric oxide.

The first prerequisite for tuning electronic states by the low dimensionality of the materials is a controlled way in altering the dimensions. One is top-down approaches where nanopatterning can be achieved by lithography or other template methods, while another option is to build the structures bottom-up with templated or nontemplated methods. Here, we utilize nontemplated growth of ZnO Q-dots with suppressed reaction speed. The growth in the nanometer region spans over minutes, hours, days, and weeks, rather than fractions of a second, which makes it possible to control the particle size with a high degree of accuracy. The particle growth was monitored in situ by measuring the band gap-dependent optical absorption edge in a UV–vis spectrometer. The Tauc plot in Figure 2 shows the band gap absorption for the solution at the start of the synthesis and for the five batches of extracted Q-dots.

In a quantum-confined particle, the optical band gap, defined by the minimum interband transition energy following electromagnetic absorption, can be described using the following equation:^12,13^
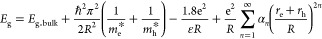
1

where *E*_g_ is the quantum-confined optical band gap, *E*_g.bulk_ is the band gap of the bulk material, *ℏ* is the reduced Planck’s constant, *R* is the radius of the particle, *m*_e_^*^ and *m*_h_^*^ are the effective masses of the electrons and holes, ε is the dielectric function, α_n_ is the polarizability, and *r*_e_ and *r*_h_ are the positions of the electrons and holes within the particle, respectively. The second term originates from an increase in kinetic energy because of localization, the third term from Coulomb attraction, and the fourth term from polarization. Neglecting higher-order terms in polarization, this expression can be simplified to a parametric form that depends on the particle diameter with
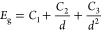
2where *d* is the particle diameter in nm, and *C*_1_*, C*_2_, and *C*_3_ are constants that previously have been experimentally determined to be 3.30, 0.293, and 3.94, respectively, for ZnO nanoparticles prepared using the same synthesis method.^13^ The particle sizes were in this way determined from the optical band gaps of the different batches of ZnO Q-dots. The batch used for the photocatalysis experiments reported here contained Q-dots with a size of 7.4 nm, and its crystal growth was stopped after 3108 min. The preparation of the photoactive electrodes was performed by depositing ZnO Q-dots on three different copper oxides: Cu_2_O, Cu_4_O_3_, and CuO, to form heterojunction bicatalysts.

To create pn-heterojunctions that enable charge transfer between p-and n-type nanoparticles, the electronic levels need to be positioned in a way that gives sufficient overlap of the available electronic states within the two materials. The electronic states of Cu_2_O and CuO are well-studied, and band positions are readily available in the literature,^14^ but for the less-investigated intermediate, copper oxide Cu_4_O_3_ less is known. For ZnO, the electronic-band energy positions depend on the particle size, and the CB position increases with decreasing particle size, whereas the VB level is constant for ZnO Q-dots in the size range used in this study.^13,15^ In Figure 2B, the absolute positions of the CB and VB are plotted for the five different sizes of ZnO Q-dots in our batches, together with Cu_2_O and CuO. The copper oxide values were taken from Jansarek et al.,^14^ and the normal hydrogen electrode (NHE) level was specified to be −4.5 V compared to the vacuum level.^16^ Here, the size-dependent CB level was measured by the potentiostatically induced Burstein–Moss shift,^15^ where the subtraction of the optically determined band gap gave the VB for the nanoparticles synthesized in this study. To be comparable, the literature data have been corrected to pH 6.6 using the Nernst equation, which, in its general form, is written as^16^
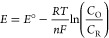
3where *R* is the ideal gas constant , *T* is the temperature in Kelvin, *n* is the number of electrons in the reaction, *F* is Faraday’s constant, and *C*_O_ and *C*_R_ are the concentrations of the oxidized and reduced species, respectively. For our specific case, eq 3 can be simplified to *E* = *E*^°^ + 0.0591 V · pH. From the determined data summarized in Figure 2, it can be concluded that the smallest ZnO Q-dots would not have sufficient electronic-state overlap with the studied copper oxides, especially with CuO, making them less suitable for this bicatalyst system. Smaller Q-dots provide a potentially higher total surface area and the ability to have a higher driving force for reduction reactions, but have, on the other hand, a higher optical band gap, resulting in lower photon absorption in the solar spectrum. Based on this, the 7.4 nm nanoparticles were chosen for making the bicatalyst samples, delivering a good compromise using a Q-dot diameter with the most appropriate CB position and, at the same time, a higher absorption of UV photons in comparison to the smaller Q-dots.

### Structural Properties

**Figure 3:**
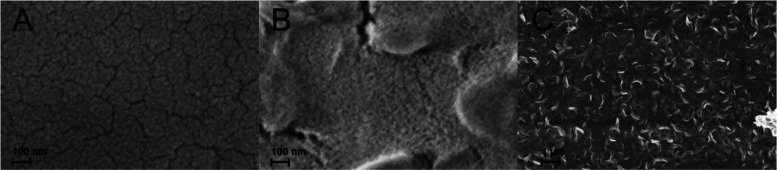
Scanning electron micrographs of (A) Cu_2_O nanoporous film with a columnar structure, (B) fine structure of ZnO nanoparticles, and (C) ZnO film in the microscale showing an even distribution of micron-sized features. The scale bars in the figures are 100 nm, 100 nm, and 1 μm, respectively.

Profilometry showed that the thickness of the sputtered copper oxides was 180 nm (Cu_2_O), 140 nm (Cu_4_O_3_), and 200 nm (CuO) and that the drop-coated ZnO Q-dot layer was about 2 μm. Scanning electron microscopy (SEM) imaging revealed a homogeneous nanoporous Cu_2_O film morphology with a columnar structure consisting of approximately 20 nm sized domains. The ZnO Q-dot layer was found to be much rougher on the nanoscale. The nanometer-sized fine structure can be seen, but there are also larger micron-sized structures and pores. Zooming out on the ZnO sample shows that the microstructures cover the surface evenly, as seen in Figure 3. The pores seen in Figure 3B could provide access to dye molecules to reach the underlying Cu_2_O.

**Figure 4:**
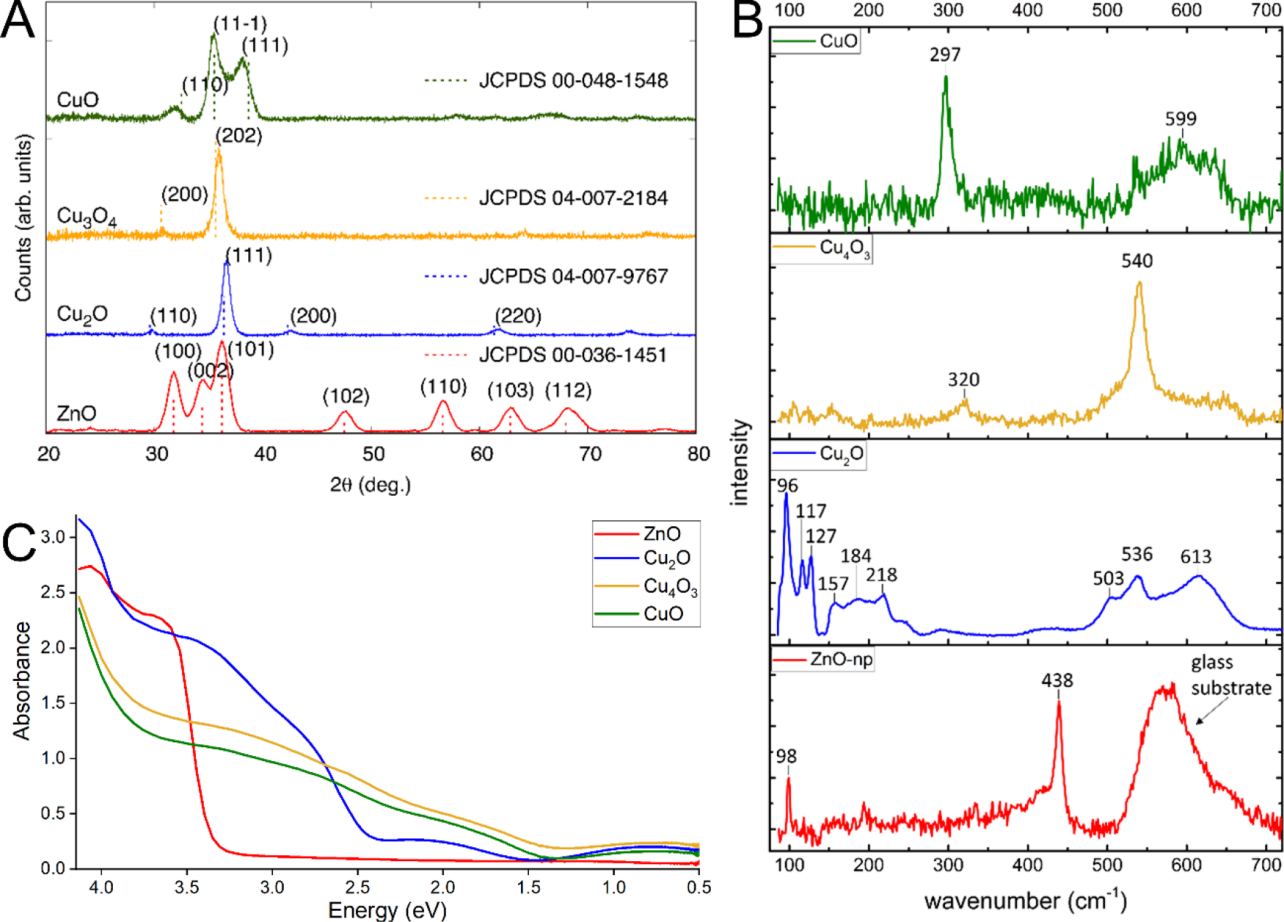
(A) XRD diffractograms from Cu_2_O, Cu_4_O_3_, CuO, and ZnO-Q-dot with peak assignments. ZnO Q-dot peaks show significant peak broadening because of the small particle size. (B) Raman spectra of Cu_2_O, Cu_4_O_3_, CuO, and ZnO-Q-dot with the peak positions marked. (C) Optical transmittance for the base materials.

Figure 4A depicts the GI-XRD diffractograms of the synthesized films, showing diffraction peaks characteristic for Cu_2_O, Cu_4_O_3_, CuO, and ZnO according to the Joint Committee on Powder Diffraction Standards (JCPDS) cards 04-007-9767 (Cu_2_O cubic), 04-007-2184 (Cu_4_O_3_, tetragonal), 00-048-1548 for the fully oxidized CuO phase (monoclinic) and 00-0036-1451 (ZnO, hexagonal) for the ZnO Q-dot film. The GI-XRD measurements verified that all materials were in the intended crystalline phases.

For the ZnO Q-dots, diffraction peak broadening analysis was performed on three different peaks using the Scherrer equation, neglecting the contribution from strain. The average crystallite size was 6.4 nm and can be compared to the value of 7.4 nm obtained from the in situ optical measurement during the synthesis, and it can be concluded that the particles from a crystallographic point of view, seem smaller, while the band gap analysis gives a slightly higher value of the particle diameter. As the Scherrer analysis is done in a solid sample and depends on how the sample is taken in the liquid container and how the solvent is removed, in comparison with the in situ optical monitoring, it is difficult to fully correlate them. The results from the X-ray diffraction (XRD) broadening, however, verify that the ZnO Q-dots are not sintered together during the deposition and annealing step and are separate crystallographic particles of the same order of size as the in situ optical measurements. The results from the different peaks are shown in Table S2 in the Supporting Information.

The Raman spectra of Cu_2_O, Cu_4_O_3_, CuO, and ZnO-Q-dot films are depicted in Figure 4B. The Raman spectroscopy measurements of the ZnO Q-dots show the position of the strongest Raman peaks (438 and 100 cm^–1^) and that the expected peaks^17^ at 333, 378, 411, and 438 cm^–1^ merge due to significant peak broadening, thus forming a broad absorption band between about 300 and the 438 cm^–1^ peak. Peak broadening in nanoparticles has previously been reported for Si^18^ and is attributed to thermal heating and size distribution of the nanoparticles. For ZnO Q-dots, several of the peaks expected from single crystal ZnO are absent because of phonon confinement from their low dimensionality.^19^ The Raman measurements confirm that the ZnO particles are present on the combined bicatalyst samples and that the different copper oxides remain in the intended phase after the Q-dot deposition and heat treatment step. It also showed that Cu_4_O_3_ exhibits a Raman signature clearly different from Cu_2_O and CuO, which confirms that it is a defined phase rather than a mixture of the other two oxides. Cu_2_O and Cu_4_O_3_ are very sensitive to laser power density and are oxidized if too much laser power is used.^20,21^ Therefore, a low laser power, 0.44 mW, at the sample was used for the measurements. Because of the orientation-dependent Raman scattering of ZnO, it is possible to investigate if the particles deposit in a preferred orientation using circularly polarized Raman spectra, according to the method recently published by our group.^17^ Large peak broadening, however, preventing the detailed analysis of the 378 cm^–1^ peak, complicated the analysis. Qualitatively, the result pointed at a tendency to the nonpolar crystal direction perpendicular to the film, but because the peak height of the 378 cm^–1^ peak had to be determined by deconvolution, the uncertainty is large, and a randomly oriented material is also possible within one standard deviation.

The UV–vis measurements in Figure 4C show that, as expected, ZnO absorbs almost all the photons with an energy higher than the band gap, whereas it is highly transparent in the visible range. The copper oxides on the other hand absorb in the visible range with an onset of the absorption λ_A_; λ_A_(Cu_3_O_4_) ≈ λ_A_(CuO) > λ_A_(Cu_2_O). The absorption of visible photons is important for efficient solar light photocatalysis. Because a lower band gap provides more photogenerated charges and less overpotential, and a high band gap material absorbs less photons while giving a higher driving force, it is important to balance them. After deposition and heat treatment, the ZnO band gap decreased to a value close to bulk ZnO. This is in qualitative agreement with the Scherrer analysis of the XRD diffraction broadening, showing that the particle size remains small but is slightly smaller than the correlated one from the optical measurements. The Scherrer analysis measures only the crystalline part of the particles and gives the volume averaged size of the crystallites.

The apparent contradiction can be due to that a surface layer of the growing ZnO Q-dots is amorphous or that there exist a small fraction of ZnO with larger diameters, but that the light absorption of this small fraction is small compared to the dominating presence of smaller particles. The size difference extracted in-between the methods, however, is quite small and is about 1 nm for the extracted diameters.

**Figure 5:**
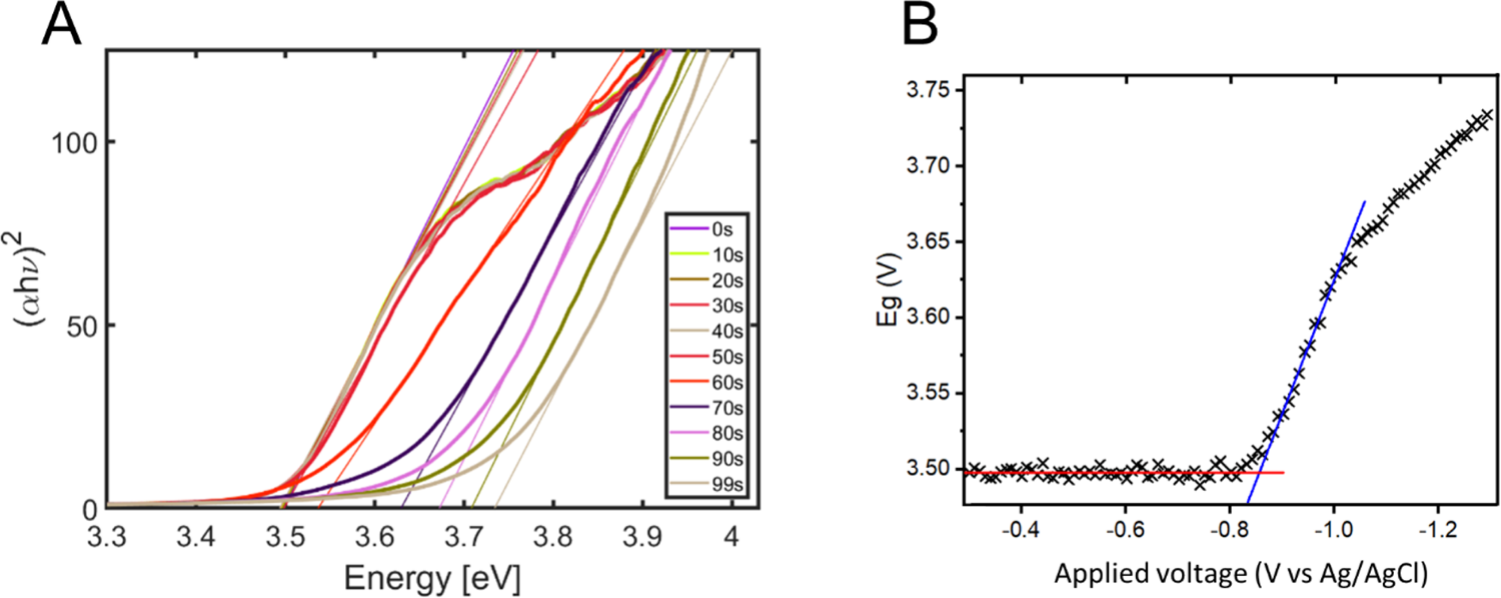
(A) Optical absorption of the ZnO Q-dots during the Burstein–Moss measurement of ZnO Q-dots with 5 nm diameter. (B) Extracted band gap values show how the optical band gap increases once the applied potential is large enough to increase the Fermi level above the CB level.

Potentiostatic Burstein–Moss shift measurements were utilized to quantify the CB edge position and showed that the apparent band gap increase occurred after an applied voltage of −0.854 V versus the Ag/AgCl reference electrode. This places the positions of the CB and VB at −0.758 and 2.744 V versus NHE, respectively. The VB level is in very good correspondence with a previous study measured under identical conditions.^15^Figure 5 shows the Burstein–Moss measurement and how the optical band gap is initially constant and then starts to increase once the Fermi level reaches the CB.

### Photocatalytic Degradation of Orange II Dye

#### Visible-Light Photodegradation

**Figure 6:**
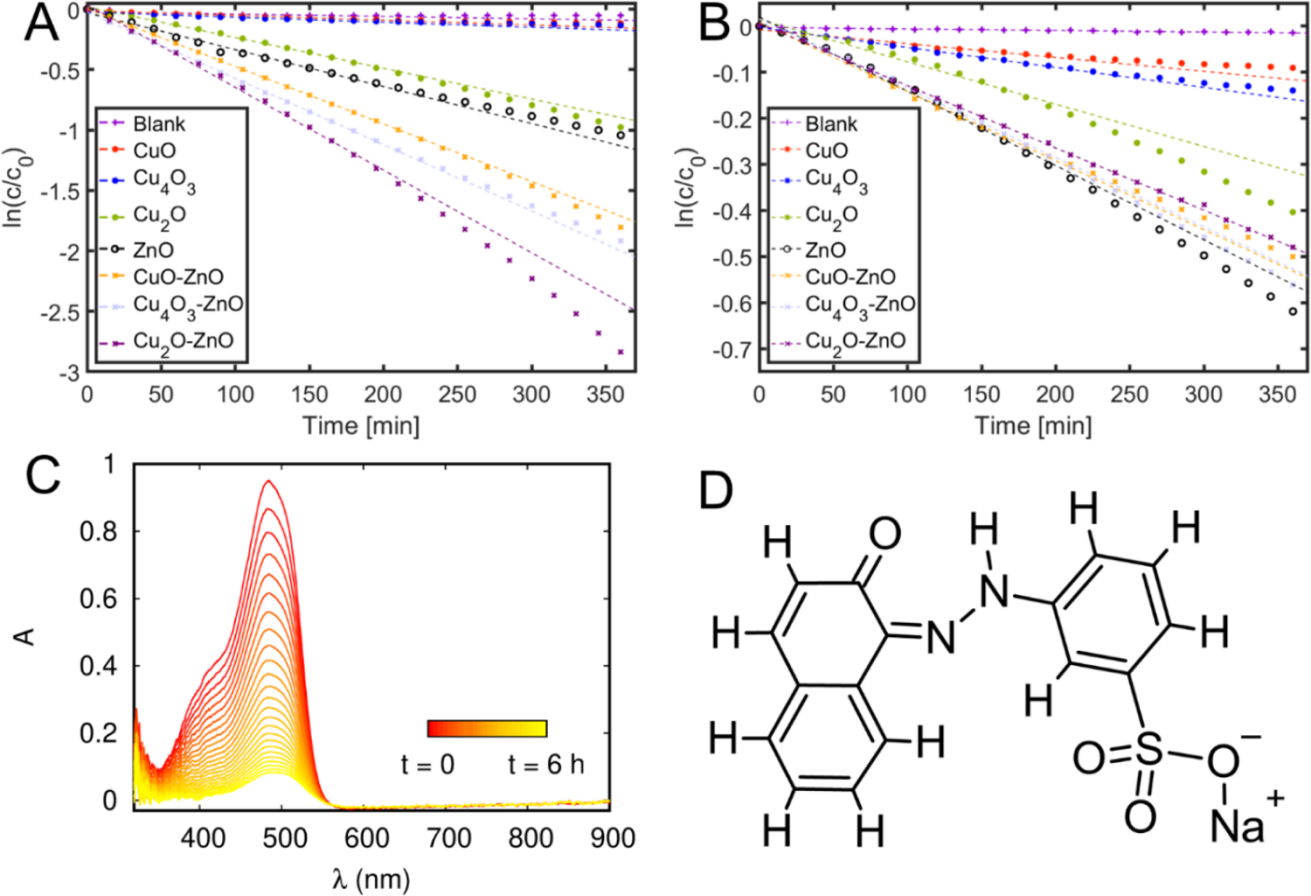
Comparison of catalyst photo removal performance under (A) visible-light illumination (405 nm) and (B) UV-light illumination (310 nm). (C) Light absorption of the orange II dye during the photo removal with vis illumination for bicatalyst Cu_2_O/ ZnO-Q-dot and (D) the structure of the orange II dye molecule. The dashed lines show the fit of the data to first-order reaction kinetics.

Although methylene blue (MB) is the most common and widespread photocatalytic probe, the use of MB is complicated by (i) substantial self-degradation under illumination, (ii) oligomerization in aqueous solution leading to metachromasy and nontrivial UV/vis band assignment, and (iii) photobleaching using a two-electron reduction process, breaking the aromaticity but otherwise leaving the molecule structurally intact.^22^ The use of orange II dye as a photocatalytic probe evade many of these effects. When studying the removal of orange II by each catalyst component independently, and under visible-light illumination, it was clear that the best-performing copper oxide was Cu_2_O. Cu_2_O removed over 60% of the dye during the experiment, Figure 6A. Here, bleaching corresponds to the photodegradation of the dye structure, losing the extended conjugation and thus the color, while a complete photodegradation would require additional reaction time. The initial photobleaching, however, is a measure of the photocatalytic performance of the different systems. The results can be compared with the optical characterization of the copper oxides (Figure 4C); Cu_2_O exhibits smaller visible-light absorption than CuO and Cu_4_O_3_, proving that Cu_2_O is intrinsically a more efficient photocatalyst for orange II degradation. For the dye removal, we have assumed first-order kinetics, and ln(*C*/*C*_0_) is plotted as a function of time in Figure 6.

In Figure 6A, it can be seen that ZnO alone removes a large proportion of the dye molecules during *visible-light* illumination. Since the energy of visible light does not exceed the band gap energy of ZnO, this cannot be due to photodegradation but is instead attributed to adsorption on the large surface on the ZnO Q-dots. On the ZnO-coated sample, coloration of the catalyst due to adsorbed dye was also observed. On the copper oxides or bicatalyst samples, this was not possible to observe because of the similar colors of the copper oxides and the dye. It was, however, verified by Raman spectroscopy that there was dye absorption also on the copper oxides.

To deposit ZnO on top of the copper oxides and not the other way around was a deliberate strategy. The light first goes through the ZnO, where the UV photons are absorbed. It is preferred that the UV photons excite ZnO, instead of the copper oxides, to be able to benefit from the high band gap of ZnO and the resulting higher driving force for degradation. The visible photons, which cannot be absorbed by ZnO, then reach the sublayer material, where they are absorbed by the copper oxide. This Z-scheme like structure greatly improves the light absorption, extending it far into the visible range, which gives a potential for improvements compared to a catalyst with only ZnO. There are, however, a number of other parameters and processes that are affected by this bicatalyst structure; some are beneficial for dye removal and some are not. We have previously mentioned how the creation of a pn-heterojunction at the interface in the bicatalyst would create an electric field, which is beneficial for the charge separation. Other effects are that the copper oxide will receive less photons compared to a standalone copper oxide catalyst because the UV photons are now filtered away and that the ZnO layer could cap and physically block the copper oxide from participating in the degradation. Combining ZnO with different copper oxides and creating bicatalysts lead to improvements for all tested combinations and the best-performing bicatalyst was the combination of Cu_2_O and ZnO, which removed 94% of the dye color from the initial photodegradation of its structure.

The formal quantum efficiency (FQE) for the different systems under vis and UV-light illumination is enlisted in Table 1. Here, the FQE is the number of decolored molecules per incident photon and can be taken as the initial efficiency to break the aromaticity in the orange II dye, while a complete degradation would require many additional steps. The photon flux from the light source was considered as ideally monochromatic with a measured photon flux *F*(λ) of 2.45 × 10^18^ photons min^–1^ m^–2^ for the 405 nm light source and 9.31 × 10^16^ photons min^–1^ m^–2^ for the 310 nm light source. The photons absorbed by the samples can be calculated using the following equation:
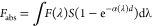
4where α(λ) is the absorption coefficient, *S* is the illuminated cross-section, and *d* is the thickness of the layer. Considering that α(310 nm) is on the order of 1.0 × 10^7^ m^–1^ and that the Q-dot layer is 2 × 10^–6^ m, all UV photons will be absorbed, while the thinner copper oxides (140–200 nm) will not have all photons absorbed at 405 nm as apparent from Figure 4C. Their absorbance at 405 nm (3.06 eV) varies from 1.0 (CuO) to 1.5 (Cu_2_O), implying that 10–3% of the photons are not absorbed. To be conservative, we still assume that all photons are absorbed in the visible light by the copper oxides when calculating the FQE. The improvement of FQE for the combined systems compared to its components is larger than an additive effect, and thus, it is a clear synergy effect. The increased photobleaching is 21% more than the summed parts of the components. The same photon flux was used for both the individual components as in the combined systems, meaning that by simply adding the component, we obtain twice the photon flux in comparison to the experiments with the combined system. Correcting for the photon flux, the improvement is instead 140% (142%). In Table 1, the photo removal coefficients and the FQE are tabulated together with the as-measured decoloration rate and rate per projected area (*A*_macro_). Here, we point out that the FQE is an apparent FQE consisting of both photodegradation and surface adsorption contributions to the change in light-absorbing dye concentration in the solution, where a general term of *photo removal* is appropriate.

**Table 1:** Photo Removal Coefficient and the FQE Calculated from the Initial 15 Data Points of the Degradation Graphs

	*k*(*t* = 0) × 10^–10^ (mol min^–1^)		*k*(*t* = 0) *A*_macro_ (μmol min^–1^ m^–2^)		FQE^a^ × 10^–5^ (molecules/incident photons )	
	VIS	UV	VIS	UV	VIS	UV
glass	0.2	0	0.2	0	0.5	0.0
CuO	0.4	0.3	0.4	0.3	1.0	19.3
Cu_4_O_3_	0.4	0.4	0.4	0.4	1.0	25.7
Cu_2_O	2.5	0.9	2.5	0.9	6.2	57.9
ZnO-Q-dot	3.1	1.6	3.1	1.6	7.6	102.9
CuO/ZnO-Q-dot	4.8	1.5	4.8	1.5	11.8	96.5
Cu_4_O_3_/ZnO-Q-dot	5.5	1.5	5.5	1.5	13.5	96.5
Cu_2_O/ZnO-Q-dot	6.8	1.4	6.8	1.4	16.7	90.0

a Apparent FQE consists of both photocatalytic degradation and adsorption contributions.

It is evident that, apart from photodegradation, adsorption is an important factor for these catalysts. The adsorption is an important step of the *direct degradation* mechanism, where the molecules need to be adsorbed to the surface in order to receive photogenerated charges from the catalyst. For chemically stable contaminants at low concentrations, it would be beneficial first to efficiently remove the contaminants from the solution by adsorption, immobilizing them on the surface of the catalyst, where they can be degraded. Adsorption is in itself a water-cleaning strategy, even in the absence of catalysis, with ZnO Q-dots acting as a nanoadsorbent.^23,24^ Comparing the visible light and UV photodegradation rate for ZnO Q-dots (Table 1), it can be seen that the photodegradation rate is larger under visible-light illumination than under UV light. This is a strong indication that the adsorption is in fact photoadsorption, that is, adsorption facilitated by the photon flux. Because the photon flux is larger in the experiment with visible light, so is adsorption. If spontaneous adsorption rather than photoadsorption were to dominate, the adsorption contribution would be very similar for the two experiments. In a parallel study with similar materials,^25^ the contributions of degradation and adsorption to the removal of dye from an aqueous solution are reported. In that study, a Cu_2_O-ZnO nanorod system was utilized with Cu_2_O as the outermost layer and evaluated using 365 nm illumination. The FQE obtained was 6.91 × 10^–5^ molecules per incident photon, which is three times lower than our results with visible light (405 nm) and about 14 times lower than our results using 310 nm. The efficiency difference highlights the importance of having a wide band gap material as the outermost material to allow for optimal spectral matching as well as the benefits of energy alignment of the two materials.

### Photodegradation Using UV Light

The results of the UV photodegradation experiments are shown in Figure 6B. Only a small difference in dye removal compared to the best-performing component was observed. Utilizing only UV light, the best-performing photocatalyst was ZnO Q-dots alone. The photocatalytic rate constants are tabulated in Table 1. Because the ZnO component sits on the top of the copper-based catalyst, it receives the same amount of light in all experiments. Therefore, a slight decrease in efficiency observed in the bicatalysts can be attributed to charge transfer from the CB of the ZnO component to the CB of the copper oxide. Because the copper oxides are less efficient in terms of photocatalytic activity than ZnO, this would result in an overall decrease in the catalytic activity of the bicatalyst. The decrease is, however, rather small, especially when compared to the improvement of performance observed for the bicatalyst under visible-light illumination.

### Photoluminescence and Charge Quenching Investigation

**Figure 7:**
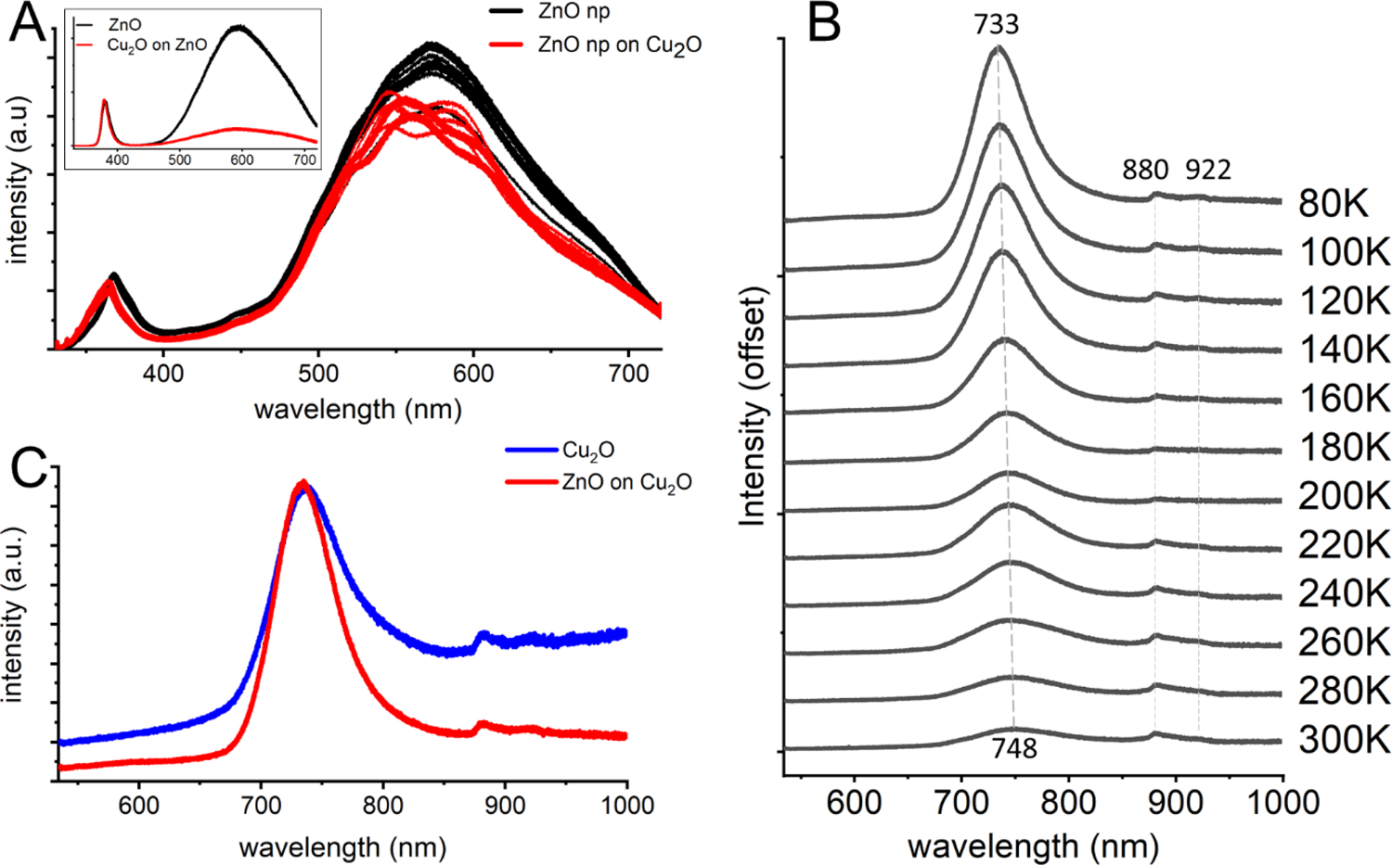
(A) PL at RT from ZnO deposited on glass (black) compared to ZnO deposited on Cu_2_O (red). The inset shows a comparison with the PL for ZnO nanorods and how a nearly complete quenching of the deep-level emission is obtained when the entire surface is coated by Cu_2_O nanoparticles. Inset data from Montero et al.^25^ (B) Temperature dependence of PL from a ZnO Q-dot /Cu_2_O film and (C) PL at 80 K from Cu_2_O with ZnO Q-dots (red) and Cu_2_O alone (blue).

Photoluminescence (PL) emission was measured for the best-performing bicatalyst. The objective was to investigate whether the electronic levels in the ZnO component changed after the creation of the heterojunction, that is, when the ZnO particles are deposited on the Cu_2_O surface. ZnO and Cu_2_O were studied in separate experiments because the study of their PL emission required the use of different excitation sources. Figure 7A shows the PL emission corresponding to ZnO Q-dot deposited on glass and ZnO Q-dot deposited on Cu_2_O. We first note that the near-band emission for the ZnO Q-dots on glass is at 368 nm, which is lower than the value of 381 nm reported for bulk ZnO.^26^ This is an expected shift from the quantum confinement effect, given the size of the Q-dots. In the case of ZnO deposited onto Cu_2_O, two distinct features emerge. First, the near-band emission is shifted to 365 nm and, secondly, the deep-level emission (500–700 nm) is reduced in intensity and assumes a different shape. The fact that the near-band emission peak shifts and broadens rather than just broadens indicates that the CB level is affected in the entire ZnO nanoparticle. The shift can be still higher in a local area near the contact point because of band bending. The deep-level emission has previously been related to intraband states caused by surface states,^27−30^ a fact that agrees with our observations: the total area of ZnO nanoparticles that are in contact with Cu_2_O is only a small fraction at the ZnO/Cu_2_O interface. Surface defects in this area can be quenched, but on the rest of the particle surface, the defect states remain. In a previous study,^25^ nanoparticles of Cu_2_O were deposited onto ZnO rods, creating the opposite situation: a fully covered ZnO surface. In that scenario, the quenching was nearly complete; see Figure 7A (inset).

The PL of Cu_2_O is rather weak at room temperature. When cooling the sample to 80 K, the main emission was increased in intensity and also shifted its peak emission from 751 to 736 nm. For the ZnO-coated Cu_2_O, the main peak was offset by 3 nm, ranging from 748 nm (300 K) to 733 nm (80 K) and also exhibited a lower background. The offset is shown in Figure 7C and the temperature dependence in Figure 7B. Again, the combination of the materials affects the PL emission. Also, there were two smaller emissions observed at 880 and 922 nm. These emissions remained there irrespective of temperature and bicatalyst formation.

**Figure 8:**
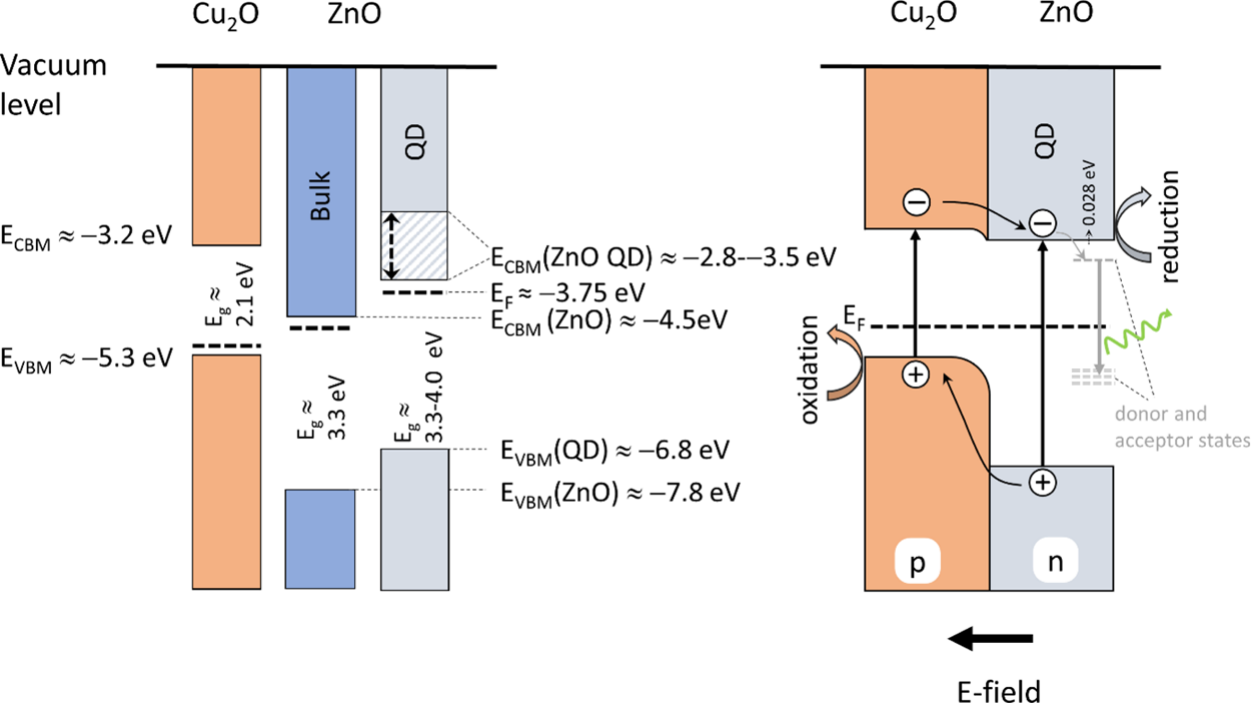
To the left, a comparison of the energy levels of the quantum-confined ZnO Q-dots and the bulk materials of ZnO and Cu_2_O and to the right, a schematic description of the pn-heterojunction.

Adding this information together, it is possible to extract the electronic levels of the ZnO Q-dots studied here and information on the interaction of the relevant energy levels of the pn-heterojunction. From the Burstein–Moss and the in situ UV–vis spectrophotometry, it is possible to determine the positions of the CB and VB for the different particle sizes. The near-band PL of ZnO moves, upon deposition onto Cu_2_O, slightly toward a shorter wavelength, indicating an increase in the band gap. It is likely that the CB moves up to better match the Cu_2_O because the unoccupied CB in the low-dimensional ZnO is more likely to move than the occupied VB. The near-band emission moves from 367.7 to 364.7 nm (energy shift Δ*E* = 0.028 eV). This is toward the Cu_2_O CB, but the adjustment is small and correspond to just slightly above 1 kT at room temperature (∼26 meV). Still, it indicates that there is a good electrical contact because the bands of the different compounds affect each other. Because of the small dimensions of the smallest Q-dots (3–4 nm), we do not expect band bending,^31^ where instead, a small adjustment of the CB is the expected outcome, while for the larger particles and the copper oxides, we expect band bending as analyzed below. The Fermi level *E*_f_ of the ZnO Q-dots at room temperature can be described with the general expression given below:
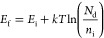
5where *E*_i_ is the intrinsic Fermi level, *k* is the Boltzmann constant, *T* is the absolute temperature, and *N*_d_ is the doping density for particles synthesized with this approach, which is around 10^19^ cm^–3^.^15^ The intrinsic carrier density *n*_i_ is given by the following equation:
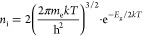
6where *m*_e_ is the mass of an electron at rest, and *E*_g_ is the bandgap. This places the Fermi level at 3.75 eV compared to the vacuum level. The green photoluminescence observed in ZnO points to the presence of intraband states. There are several possible causes for these states: Zn vacancies, Zn interstitials, antisite oxygens, and oxygen vacancies, where the latter has been argued to be the most likely cause.^13^ This conclusion is strengthened by our observation that the ZnO deep-level PL emission is quenched upon contact between ZnO and Cu_2_O and that the quenching is higher when a larger proportion of the ZnO surface is in contact with Cu_2_O. The upper transition level for the fluorescent states in ZnO Q-dots has been determined to be at 0.35 eV below the CB by spectroelectrochemical measurements.^30^ The acceptor states for the fluorescence are not potentiostatically available but, according to our PL measurements, they are located 2.1–2.34 eV further down. Figure 8 summarizes the band gap and energy levels of the ZnO Q-dots in comparison with the bulk ZnO and Cu_2_O. For charges that are transferred across the pn-heterojunction, reduction reactions are likely to take place on the ZnO and oxidation reactions on Cu_2_O, although part of oxidation reactions on ZnO and reduction at Cu_2_O cannot be excluded from charge carriers that are not transferred.

For a quantitative analysis and choice of the size of the n-type Q-dots to have a sufficiently good energy alignment with different copper oxides, energy-level characterization of the individual parts is sufficient. For a more detailed picture, also the varying degree of band bending, Fermi-level pinning, and interface properties have to be taken into account. Utilizing the linearized Poisson–Boltzmann equation, the potential difference (Δϕ) of the center (*r* = 0) of a spherical semiconductor particle with respect to the potential at a different position (*r*) can be written as follows:^32,33^
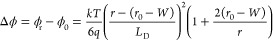
7where *r*_0_ is the radius of the particle, *r* is the position of the evaluated potential with respect to the center, *k* is the Boltzmann constant, *T* is the temperature, *q* is the elementary charge, *W* is the width of the space charge layer, and *L*_D_ = (ε_0_ε*kT*/2*q*^2^*N*_D_)^1/2^ is the Debye length expressed as a function dielectric properties, ε, and the number of ionized dopants *N*_d_ in the semiconductor. For large particles (*r*_0_ > *W*), eq 7 reduces to Δϕ = (*kT*/2*q*)(*W/L*_D_)^2^ and is valid for larger ZnO particles and the planar copper oxide layers, while in the limit of small Q-dot particles (*r*_0_ < *W*), one can obtain the following relation:
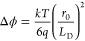
8

Considering the Debye length at a constant temperature and unaltered dielectric properties of the semiconductor material, the remaining determining factor for the potential drop is the number of ionized donors, *N*_d_, in the material and the radius of the particle from eq 8. Using a dielectric constant ε = 7.9 for the ZnO, considering the situation at room temperature (298 K) and using the aforementioned doping density of 1 × 10^19^ cm^–3^, we obtain a potential difference between the center and the surface of a 7 nm diameter ZnO Q-dot of 93 mV, while a 3 nm Q-dot would have 17 mV potential drop and thus negligible band bending. If the particles are too small to develop a space charge layer during illumination and charge-carrier transfer to the desired redox species in the solution, the electric potential drop will instead occur in the Helmholtz layer with a subsequent shift of the position of the band edges.

For a quantitative analysis of Q-dot sizes matching energy levels in different copper oxides or other materials, analysis of the energy levels in isolated Q-dots is sufficient. For a more detailed analysis, the precise amount of ionized donors and the amount of band bending in the differently sized Q-dots have to be quantified, and likely also the electronic structure at the interface. The latter would be important as any remaining defect states in the ZnO/copper-oxide interface could form chargeable states and thus contribute to Fermi-level pinning and thus altering the band-bending situation.

## Conclusions

We report the ability to control electronic states and energy alignment by utilizing size-controlled quantum dots (Q-dots) of ZnO in combination with three copper oxides: Cu_2_O, Cu_4_O_3_, and CuO. The band gap and energy-level shifting from the quantum confinement effects in the ZnO nanoparticles in the diameter range from 2.6 to 7.4 nm and a change in the optical band gap from 3.99 to 3.41 eV are reported together with the possibility to utilize this to align the electronic bands in heterojunction photocatalysts to provide an improved efficiency. The most promising bicatalyst based on energy-level alignment analysis was the combination of the ZnO-Q-dot of 7 nm diameter and Cu_2_O. This combination showed a 140% higher dye photo removal with visible light compared to the single components. For full characterization, averaging over more identical samples, and optimization of the exact Q-dots size in this system for maximum performance with respective copper oxide, further experiments are needed. The synergetic effect together with PL measurements indicates a sufficiently good physical and electrical contact between ZnO and Cu_2_O, where the improved performance likely can be attributed to the formation of a heterojunction with well-aligned conduction-band levels in-between the materials. Similar energy-level tuning is anticipated to be of use for targeting overpotential-dependent reduction reactions or extended other material compositions, as long as at least one of the materials in the heterojunction can be electronically tuned via quantum confinement.

## Methods

### Material Synthesis

The copper oxides were prepared by reactive DC magnetron sputtering using a Balzers UTT 400 sputter unit with a copper target operated in the reactive sputtering mode in the nanoporous region of the Thornton diagram. The composition of the copper oxide film was controlled by regulating the oxygen flow into the sputtering chamber. The deposition process follows the procedure of a previous publication^34^ and yields phase-pure nanoporous films with the usual columnar structure. The sputtering parameters for the three copper oxides are listed in Table 2.

**Table 2:** Process Parameters for the Magnetron Sputtering of the Copper Oxides and the Thickness of the Samples Selected for the Study

copper oxide phase	oxygen flow (sccm)	discharge power (W)	argon flux (sccm)	discharge current (mA)	total pressure (mtorr)	thickness (nm)
Cu_2_O	6.00	250	50.0	444	31.8	180
Cu_4_O_3_	10.00	250	50.0	472	33.4	140
CuO	14	250	50.0	470	34.3	200

The ZnO Q-dots were synthesized using a wet chemical method based on the work of Spanhel et al.^27^ and Meulenkamp,^35^ which have been slightly modified. A detailed description of the ZnO Q-dots synthesis is included in the Supporting Information. In short, two saturated solutions of lithium hydroxide and zinc acetate in ethanol were prepared. These compounds are precursors for ZnO and when the two solutions are mixed together, the precursors react according to reaction 1,
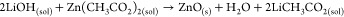
R1creating a supersaturated solution of ZnO. Upon mixing, the nucleation of ZnO Q-dots is instantaneous, and the ZnO Q-dots start to grow. One major advantage of this synthesis lies in the relatively slow kinetics of the growth reaction. A sample from the solution was placed in the spectrometer immediately after the mixing of the precursor solutions, and the progress of the Q-dot growth could in that way be monitored in real time. At five specific times, a part of the ZnO Q-dot solution was extracted from the reaction flask, and the nanoparticles were purified with the following washing procedure: 15–20 mL of ZnO Q-dot solution was retrieved from the reaction flask, and hexane was added until the solution turned cloudy. The solution was centrifuged at 5000 rpm for 3 min, and the supernatant was subsequently removed. The ZnO Q-dots were then redispersed in approximately 10 mL of methanol (48–50 drops per centrifugation tube). The ZnO Q-dots grow continuously in the reaction vessel with a comparably fast growth in the beginning. This means that the extraction procedure time is not negligible, and it has been assumed that the time of the solvent exchange, when ZnO Q-dots aggregate and precipitate, defines when the particles stop growing. The Tauc plot in Figure 2 shows the band gap absorption for the solution at the start of the synthesis for five batches of extracted Q-dots.

Bicatalyst samples using ZnO Q-dots in combination with the three copper oxides were prepared. To be able to show synergy effects, positive or negative, from the combination of the photocatalytic materials, samples with single catalyst materials of Cu_2_O, Cu_4_O_3_, CuO, ZnO Q-dot and a glass substrate reference were prepared for comparison. Microscope slide glass was used as substrates for all samples. Before the sample preparation, the substrates were cleaned with water and detergent, rinsed with water, rinsed in ethanol, sonicated in ethanol in an ultrasonic bath for 60 min and dried in air. Before the application of ZnO Q-dots on the substrates, the ZnO Q-dot solutions were ultrasonicated for 30 min to disperse the Q-dots, and the substrates and samples were treated with UV/ozone for 20 min to activate the surface and increase the surface adhesion. The samples without ZnO Q-dots were also subjected to the same UV ozone treatment to ensure consistency. One drop of methanol solution with ZnO Q-dots was drop-coated onto a 10 mm × 10 mm substrate that was maintained at 40 °C on a hotplate. The drop spread evenly across the sample, and the solvent evaporated rapidly. The samples were then heat-treated in a furnace (Logotherm, Nabertherm) at 250 °C for 20 min, to improve the adhesion. An overview of the different samples, material combinations, and the sample preparation process is shown in the Supporting Information Table S1.

### Material Characterization

The film thickness characterizations of the sputtered copper oxide films and the drop-coated ZnO Q-dot films were performed using a touch probe profilometer (Bruker Dektak XT). SEM was performed with a Zeiss LEO 1530 SEM instrument using 5000 V acceleration voltage. Grazing incidence XRD (GI-XRD) was performed on the three copper oxide films for phase identification and on films deposited with ZnO nanoparticles using a Siemens D5000 Kristalloflex diffractometer with a parallel beam geometry using Cu K_α_ radiation (λ = 1.5418 Å). The angle of incidence was fixed to 1°. Grain size determination was done by Scherrer analysis.

In situ UV–vis spectroscopy measurements were carried out to monitor the crystal growth process during the ZnO Q-dot synthesis. A fiber optical spectrometer (Ocean optics HR2000+) was used together with a combined halogen and deuterium light source (Mikropack, DH-2000-BAL) to measure the optical absorption from a sample of the growth solution placed in a 10 mm × 1 mm PMMA cuvette. One measurement per minute was taken for 24 h, and the measurement settings used were 1 ms acquisition time, averaging five spectra.

Burstein–Moss shift measurement was performed at room temperature in a spectroelectrochemical setup to determine the absolute positions of the CB and VB for the ZnO Q-dots. For this measurement, ZnO particles were deposited by doctor-blading on a glass coated with a fluorine-doped tin oxide (SnO_2_:F, sheet resistance of 8 Ω/square, TEC8, Pilkington). A three-electrode measurement was set up using aqueous 0.5 M Na_2_SO_4_ as buffer solution, Pt as the working electrode, and Ag/AgCl as the reference electrode. A potential from −0.3 V to −1.3 V vs Ag/AgCl was applied to the sample using a potentiostat (CH-instruments, 760C workstation), the sweep rate was 10 mV/s, and the optical absorption of the film was measured in situ every second using the abovementioned fiber-optic UV–vis setup.

Raman measurements were performed using a micro Raman spectrometer (Renishaw, Invia Reflex) with a 532 nm laser, a 2400 lines/mm grating, and a 100× Leica N-plan objective. For the characterization of the various catalyst samples, the acquisition time was 60 s and the number of accumulations was 360, giving a total measurement time of 6 h. The laser power at the sample was limited to 0.44 mW. Automatic cosmic ray removal was used. The unusually long measurement time is due to the fact that the thermal instability of the copper oxides prevented the use of higher laser power. Additional measurements were performed on ZnO to investigate the Q-dot orientation using circularly polarized light, 44 mW power at the sample, and a total measurement of 60 × 10 s. UV–vis spectroscopy was used for the optical characterization of the copper oxides and the ZnO Q-dots after application using a spectrometer (Lambda 900, Perkin Elmer) with an integrating sphere. Transmittance was measured for the wavelength range of 300–2500 nm.

PL spectroscopy was used to measure the near-bandgap emission and deep-level emission of the investigated samples. High-resolution Raman spectrometers (Renishaw, Invia Reflex) employing 325 nm (for ZnO) and 532 nm (for Cu_2_O) laser excitation sources were utilized for the PL measurements. The calibration was verified with diamond and silicon reference samples. For UV excitation, a 2400 lines/mm grating and a 40× NUV objective with NA = 0.47 were used. Data were acquired for 10 s with 0.1 mW laser power at the sample. For vis excitation, a 1200 lines/mm grating and a 50× LWD objective with NA = 0.5 were used. Data were acquired for 10 s with 0.44 mW laser power at the sample. A liquid-nitrogen-cooled sample stage (HFS600E Linkam Scientific instruments) was used to control the temperature from 80 to 300 K. The spectrometer used an extended scan utility enabling continuous acquisition of the full spectral range, which was 330–720 nm for UV and 535–1000 nm for the vis measurements.

The photocatalytic experiments were performed in a setup, allowing for simultaneous in situ UV–vis spectrometry to monitor the removal of model dye pollutants. Photocatalytic dye degradation experiments were carried out using both visible light (405 nm, 25.5 mW/cm^2^) and UV light (310 nm, 1.27 mW/cm^2^) using a multiple output diode light source (Prizmatix, FC5-LED). The samples were illuminated using optical fibers, and the photon power specified was measured at the position of the sample using a pyranometer (Thorlabs, PM160T). For data acquisition, a fiber optical spectrometer (Ocean optics, HR400CG-UV-NIR) was used together with a halogen light source (Ocean optics, HL-2000-FHSA). The samples were placed in cuvettes, 10 mm × 10 mm with all sides clear, toward the back of the cuvette. PMMA cuvettes were used for the visible-light photo removal and quartz cuvettes for the UV-light photo removal. An amount of 2 mL of 50 μM of orange II solution in H_2_O was added to the cuvette. The experiment was started by simultaneously opening the light source shutter and starting the data acquisition. Spectra were acquired every 15 min for 6 h, and the measurement settings used were 20 ms acquisition time, averaging over 20 spectra for both the visible-light and UV-light degradation experiments. All phototcatalysts were tested for both visible and UV-light photocatalytic dye degradation.

## Supporting Information Available

More detailed information about the synthesis and sample treatment scheme. Details from the Scherrer analysis (PDF)
