# Supplementary material for: Energy Alignment of Quantum-Confined ZnO Particles with Copper Oxides for Heterojunctions with Improved Photocatalytic Performance
Source: ACS Nanosci Au. 2021 Dec 21;2(2):128–39. doi: 10.1021/acsnanoscienceau.1c00040 (PMC10125148; doi:10.1021/acsnanoscienceau.1c00040)
Supplement: Supplementary file 1 — ng1c00040_si_001.pdf [file ng1c00040_si_001.pdf]

Supporting information

Energy-alignment of Quantum Confined ZnO particles with Copper Oxides for Heterojunctions with Improved Photocatalytic Performance

Jakob Thyr, José Montero, Lars Österlund, and Tomas Edvinsson*

Department of Materials Science and Engineering, Division of Solid State Physics
Uppsala University, P.O. box 35, SE 75103 Uppsala, Sweden.

KEYWORDS: quantum confinement, ZnO quantum dots, copper oxide, energy-alignment, heterojunctions, photocatalysis.

ABSTRACT: The ability to control electronic states by utilizing quantum confinement of one of the material components in heterojunctions is a promising approach to perform energy level matching. In this work we report the possibility to achieve optimum energy-alignment in heterojunctions made from size-controlled quantum dots (Q-dots) of ZnO in combination with three copper oxides: Cu_2O, Cu_4O_3 and CuO. Quantum confinement effects in the ZnO nanoparticles in the diameter range 2.6 nm to 7.4 nm showed that the direct optical band gap decreased from 3.99 eV to 3.41 eV, with a dominating shift occurring in the conduction band (CB) edge, and thus the possibility to obtain close to 0.6 eV CB edge shift by controlling the size of ZnO. The effect was utilized to align the electronic bands in the ZnO Q-dot/copper oxide heterojunctions to allow charge transfer in-between the materials, and to test the ability to improve the photocatalytic performance for the system. The catalyst materials were investigated with X-ray diffraction, scanning electron microscopy, UV-Vis-, photoluminescence-, and Raman spectroscopy. The most promising material combination was found to be the Cu_2O copper oxide in combination with an energy aligned ZnO Q-dot system with approximately 7 nm diameter, showing strong synergy effects in good agreement with the energy level analysis, outperforming the added effect of its individual components, ZnO-Q-dots and Cu_2O, by about 140%. The results show that utilization of a heterojunction with controllable energy-alignment can provide a drastically improved photocatalytic performance. Apart from increased photocatalytic activity, specific surface states of ZnO are quenched when the heterojunction is created. It is anticipated that the same approach can be utilized in several material combination with the added benefit of a system with controllable overpotential for the reduction reaction and thus added specify for the targeted reduction reaction.

METHODS

Material synthesis

As precursors for ZnO $LiOH·H_2O$ (Sigma-Aldrich, 99.95%) and $Zn(Ac)_2·2H_20$ (Fluka, 99.5%) was used. The solvents and extraction media used were ethanol (VWR AnalaR NORMAPUR, 99.8%), methanol (VWR AnalaR NORMAPUR, 99.8%) and n-hexane (VWR AnalaR NORMAPUR, 99%). The precursors were weighed on an analysis scale (Ohaus model PA114C).

Both precursor solutions were prepared simultaneously but are for clarity reasons here described one after another. A LiOH solution was prepared in a 250 ml round flask filled with nitrogen gas to create a non-oxidizing atmosphere. 0.290g LiOHxH2O was weighed and added to the flask. 50mL of ethanol was added. The flask was inserted in a water bath for heating and a stirring magnet with a rotation speed of 100rpm was added to the flask. A light flow of nitrogen gas was used to purge the vessel for 5 min after which the nitrogen flow was stopped and the bottle was sealed with para film. The ethanol level was marked on the bottle to keep track of solvent evaporation. The water bath heater was set to low level of heating (50-60 degrees) to slowly increase the temperature of the water bath, an estimated final temperature reached was 40°C. Some solvent evaporation occurred during the dissolution process and a total of 7 mL ethanol was added to keep the initial level. After 1h 24min almost all of the lithium salt had been dissolved. The few remaining salt crystals were filtered out using a paper filter, resulting in a clear solution. The Zn(Ac)2 solution was prepared in a double necked round flask, where one neck was used to add the solvent and salt and was otherwise sealed and the other one was fitted with a water cooled Dimroth condenser to return evaporated ethanol to the flask during the experiment. 45mL of ethanol and a stirring magnet was added to the flask, the flask was sealed and heated in a water bath until the ethanol started to boil. 1.100g of $Zn(Ac)_2$ was weighed and added to the boiling ethanol under vigorous stirring. After 2minutes the $Zn(Ac)_2$ salt had dissolved and the flask was cooled in an ice bath.

Sample treatment scheme

An overview of the studied samples, how they are built up and the different treatment procedures they were subjected to are shown in table S1.

Table S1. The schematic layer structure and preparation procedures for the samples investigated in the study.

	**Reference**	**ZnO**	**Cu_2O**	**Cu_2O bicatalyst**	**Cu_4O_3**	**Cu_4O_3 bicatalyst**	**CuO**	**CuO bicatalyst**
Layer 2				ZnO np		ZnO np		ZnO np
Layer 1		ZnO np	Cu_2O	Cu_2O	Cu_4O_3	Cu_4O_3	CuO	CuO
Substrate	Glass	Glass	Glass	Glass	Glass	Glass	Glass	Glass
Substrate cleaning*	Yes	Yes	Yes	Yes	Yes	Yes	Yes	Yes
Sputtering			Cu_2O	Cu_2O	Cu_4O_3	Cu_4O_3	CuO	CuO
Activation	UV/Ozone 20min	UV/Ozone 20min	UV/Ozone 20min	UV/Ozone 20min	UV/Ozone 20min	UV/Ozone 20min	UV/Ozone 20min	UV/Ozone 20min
Particle dispersion		30min		30min		30min		30min
Application		Dripcoating 1drop (50uL) substrate at 40C		Dripcoating 1 drop (50uL) substrate at 40 C		Dripcoating 1 drop (50uL) substrate at 40 C		Dripcoating 1 drop (50uL) substrate at 40 C
Heat treatment	250C, 20 min	250C, 20 min	250C, 20 min	250C, 20 min	250C, 20 min	250C, 20 min	250C, 20 min	250C, 20 min

* Cleaning procedure: Water and dishwashing detergent; Rinse with water; Rinse with ethnol; Sonicate in ethanol in ultrasonic bath for 60min; Rince with water; Dry in air.

RESULTS

Scherrer analysis

Scherrer analysis was done on three peaks of the ZnO np diffractogram with similar results. The results are shown in table S2

Table S2. Crystallite sizes obtained when applying the Scherrer-equation to three peaks of the ZnO diffractogram.

Peak	Inst. broadening	Crystalite Size (Å)
47.6	0.284	63.6
56.7	0.284	65.9
62.9	0.30	63.0
